# Parafoveal preview benefit in a conflicting sentential context: Evidence from ERPs

**DOI:** 10.3389/fpsyg.2022.1063923

**Published:** 2022-11-15

**Authors:** Nan Li, Gantang Li, Suiping Wang

**Affiliations:** ^1^School of Foreign Studies, South China Normal University, Guangzhou, China; ^2^Philosophy and Social Science Laboratory of Reading and Development in Children and Adolescents (South China Normal University) Ministry of Education, Guangzhou, China; ^3^Center for Language Cognition and Assessment, Guangdong, China; ^4^School of Psychology, South China Normal University, Guangzhou, China

**Keywords:** identity preview benefit, plausibility, ERP, N400, Chinese reading

## Abstract

In natural reading, the reader’s processing of a word starts when the word is located in parafoveal vision. Relative to a situation with an invalid preview, fixations are significantly shorter after a fully valid, identical preview. Although research on the identity preview benefit has been ongoing for more than 40 years, the mechanism of this preview benefit, the level of lexical processing it occurs, and its relationship to the sentential context remain unclear. This study employed EEG brain component analysis technology to address these questions in Chinese sentence reading. We manipulated the sentential context to make the target word plausible or not plausible with the sentence and manipulated the target word present or not present in preview. EEG results showed that the identity preview benefit can affect not only the early preview positivity, reflecting the early orthographic processing of words, but also the N400 and LPC components, reflecting the late and in-depth semantic processing of words. Conflicting sentential context, in which the target word is implausible and cannot be integrated into the sentence, can interfere temporarily with these processes. These findings suggest that in the process of sentence reading, an identical preview word can promote the subsequent reading process at multiple levels, and its role is modulated by contextual information.

## Introduction

During reading, readers move their eyes in order to bring new words into foveal vison, where visual acuity is the highest. There is less visual acuity as the eyes move from foveal to parafoveal vision, and the amount of information gleaned from parafoveal is limited (Rayner, 1998). Nevertheless, information obtained from the parafovea can make reading more efficient (McConkie and Rayner, 1975; Rayner, 1975). One of the key questions in this area of research concerns how this preview processing influences upcoming foveal recognition (Schotter et al., 2012). This study focuses on how the reader’s preview processing of a word facilitates the subsequent processing of the same word in direct fixation, and how this preview benefit is modulated by contextual information provided by the sentence.

Parafoveal processing commonly is investigated by using either eye movements (EM) with a boundary paradigm (Rayner, 1975) and event related potentials (ERP) with an RSVP-with-flankers paradigm (Barber et al., 2010). In the boundary paradigm, a sentence is presented naturally and readers commonly conduct left-to-right reading based on the language script. An upcoming word in a sentence is masked while in parafoveal vision and only unmasked once it receives a direct fixation. The strongest facilitation is observed after a fully valid, identical preview, which is typically called the identity preview benefit. To obtain the EEG correlates of identity preview benefit, however, it is methodologically challenging because EEG recordings are very sensitive to artifacts caused by eye-movements. Co-registration of EEG and eye-tracking measures with boundary paradigm (free of eye movements) raises several methodological difficulties, such as disentangle ocular artifacts and overlapping signals (Dimigen et al., 2011). Therefore, RSVP-with-flankers paradigm, a modification of the canonical word-by-word sentence presentation (i.e., RSVP) procedure, is applied in some of the EEG studies of preview effect. In this paradigm, to avoid eye movements, sentences are presented as word triplets during steady fixation. The preceding word in the sentence shown to the left of fixation and the subsequent word is shown to the right of fixation. The identity preview benefit can be measured by comparing the EEG amplitude elicited by the foveal word after an identical preview and after an uninformative/invalid preview. While RSVP-with-flankers paradigm is undoubtedly different from natural reading (Sereno and Rayner, 2003; Dimigen et al., 2011), it avoids most of the methodological challenges related to EEG recording with eye-movements and allows to study the brain-electric correlates of preview effect in reading.

Readers always see a correct (identical) preview on the upcoming word during natural reading. Thus, the effect of processing an identical preview word on the processing of the same word in direct fixation can be seen as a special kind of repetitive priming effect. In this type of priming, the processing of the priming word occurs in the preview vision, and the processing of the target word occurs in the subsequent direct fixation. The effect of processing of an identical preview word can be revealed by the nature of this priming effect. Previous studies on foveal word priming (priming by a previously fixated word) demonstrate that a word’s repetition triggers a cascade of processes, including visual feature representations, such as fonts or positions (P1/N1), sub-lexical processing, such as mapping of abstract orthographic or phonological information (N250, with orthographic influence in the early phase and phonological influence in the late phase. But see in the studies of the time course of visual word recognition, orthographic process occurs earlier and modulates the N150 or N170 components; e.g., Bentin et al., 1999; Maurer et al., 2005; Grainger et al., 2006; Grainger and Holcomb, 2009), implicit lexico-semantic processing (N400; e.g., Rugg, 1985; Van Petten et al., 1991; Holcomb and Grainger, 2007; Grainger and Holcomb, 2009), and explicit recall of the prior presentation or elaborative contextual updating (LPC; e.g., Rugg, 1985; Besson et al., 1992; Besson and Kutas, 1993; Rugg et al., 1998).

If repetition of a previously fixated word triggers a cascade of processes, then repetition of preview word may involve similar processes. The results of some studies are consistent with this view (e.g., Dimigen et al., 2012; Li et al., 2015; Antúnez et al., 2021; Dimigen and Ehinger, 2021; Li et al., 2022). For example, Dimigen et al. (2012) found effect of repetition of a word from preview beginning after the N1 peak, reaching a maximum around 250 ms in occipitotemporal cortex. This decreased negativity to valid previewed words was called preview positivity. The time range and topography of this preview positivity reflects priming at the level of abstract orthographic representations and may be functionally related to the early phase of N250 component (Grainger et al., 2006; Dimigen et al., 2012). In addition, repetition a word from preview also reduced the size of the following N400 component, which was attributed to lexico-semantic processing (e.g., Dimigen and Ehinger, 2021). This shows the time range and topography of the repetition effect is compatible with those in fovea priming studies. This suggests that there is a commonality between the repetition effect of a word in preview and the repetition effect of a word in previous fixation, and each can occur in multiple time windows at multiple levels of lexical processing.

Most models of the repetition effect propose that the initial presentation of a word changes the activation level of the word, facilitating word processing at the time of the second presentation (the abstractionist view; Carr et al., 1994; Tenpenny, 1995). However, word priming in context may arise from mechanisms that are outside of the lexicon. These mechanisms may be determined by the activation of elements that are relevant to the discourse (discourse-based perspectives, Foss and Speer, 1991) or by memories of prior episodes with a given word (the episodic view; Levy and Burns, 1990; Masson and Michael, 1993). This contextual effect in word priming is usually measured by the contextual plausibility. It refers to whether a word is a reasonable and likely continuation of the ongoing sentence. It can reflect consistence between the word and the context. In a conflicting sentential context, the meaning of the sentence (up to and not including the target word) does not fit the meaning of the target word, resulting a failure of integrating the target word into the sentential context. However, in the foveal priming studies, it is shown that a conflicting context does not disturb a word’s repetition effect (e.g., Besson et al., 1990, 1992; Lai et al., 2021). This suggests that the repetition effect may be independent of the sentential context.

It is worth noting that in foveal priming studies (priming of a fixated word), there is often a long interval (a few words to several sentences) between the priming word and the target word. It is possible that the impact of contextual conflict cannot survive from repetition with long interval. That is, it is possible that the impact of contextual conflict can not be detected when the repetition starts a few words after fixating the prime word. In contrast, the time interval between the processing of a prime word in preview and the processing of the target word in fovea is very short (within 20–30 ms saccade). Thus, the priming happens instantly after the processing of the priming word. Studying the preview repetition effect (identity preview effect) is a useful way to test the role of context in the instant priming process. This can be accomplished by testing whether a conflicting sentential context can indeed hinder the immediate preview benefit on the target word.

Few studies have tested the impact of context on the repetition (identity) preview effect. Several studies have reported the impact of contextual expectation on the identity preview benefit using eye-tracking technology (Balota et al., 1985; Choi et al., 2017; Veldre and Andrews, 2018). The results showed that highly expected words produced a larger identity preview benefit (shorter fixation duration) than unexpected words. However, whereas plausibility can reflect the conflict or consistence between the word and the context, expectation can only reflect the degree of lexical information of word activated by context. In addition, eye movement measures assess the minimum information necessary to initiate the next saccade, and they reflect the end product and summation of cognitive processes. Thus, it may be more promising to measure EEG when to test, the online processing of different levels of information.

To our knowledge, only one EEG study using Chinese materials has addressed the question of whether a word’s plausibility modulates the identity preview benefit (Li et al., 2022). However, because the main purpose of that study was to investigate the interaction of preview plausibility and foveal plausibility on foveal word processing, there was relatively limited manipulation of repetition priming. Specifically, the study design required that the preview word and foveal word be either plausible words or implausible words. For example, in their sentence “*The students hid in the **cave** in the mountain to avoid wind and rain*” (同学们躲进山上的那个洞来躲避风雨), the target word at the preview position was presented as either plausible with context (cave) or implausible with context (egg). The target word in the foveal position was presented either in its identical (cave/egg), plausible (pavilion), or implausible version (cup). The orthogonal manipulation disclosed an interaction between the preview and foveal words’ plausibility.

By manipulation, they showed the foveal N400 amplitude was separately modulated by both word identity and plausibility. That is, there is no negative impact of contextual conflict on the identity preview benefit. One possibility is that identity preview benefit is independent of the contextual conflict. However, it may also be possible that the impact of contextual conflict under the identical preview condition differs from that under the uninformative/invalid preview condition. For example, the contextual conflict may cause more interference under the condition of uninformative/invalid previews, and cause less interference under the condition of identical previews. To test these possibilities, it is valuable to control the contextual information provided by the uninformative/invalid previews, so to minimize the contextual effect under the uninformative/invalid preview condition.

We propose that one way of controlling the contextual information provided by the uninformative/invalid previews is to use pseudowords. Because pseudowords have neither lexical information nor contextual information, the interference of contextual conflicts under the condition of uninformative/invalid previews can be minimized. Thus, the comparison between the identity vs. uninformative/invalid preview conditions can only reflect the identity preview benefit and the interference caused by the contextual conflict under the identity preview condition.

In this study, we conducted an ERP experiment with Chinese readers using the RSVP-with-flankers paradigm and rigorous fixation control *via* eye tracking. We examined how the identical preview word affects the processing of the target word in fixation. We addressed two questions. First, how does the preview of the target word affect the foveal processing of the target word in the early processing stage of orthography (preview positivity) and late processing stage of semantics (N400, LPC)? Second, does contextual conflict affect these preview repetition processes? To answer these questions, we manipulated the sentential context to make the target word plausible or not plausible with the sentence, and manipulated the target word present or not present in preview. Specifically, there were four conditions: (1) plausible context & identical preview; (2) implausible context & identical preview; (3) plausible context & invalid preview; (4) implausible context & invalid preview. We used pseudowords as uninformative/invalid previews. Identity preview benefit was measured by comparing conditions (3) and (4) versus conditions (1) and (2). From previous findings (e.g., Dimigen et al., 2012; Dimigen and Ehinger, 2021), we expect the identical preview can strongly facilitates foveal word processing, and trigger benefits through a cascade of effects of the foveal word on the early preview positivity, or even the late components of N400 and LPC. By comparing the identity preview benefit when the preview words are plausible (condition 3 versus condition 1) with the identity preview benefit when the preview words are implausible (condition 4 versus condition 2), we can examine the relationship between the identity preview benefit and the contextual plausibility. If context can influence the instant priming process, we expect to see a negative impact of contextual conflict on the identity preview benefit. Since the contextual conflict reflects the processing of the meaning of sentence, we expect contextual conflict is more likely to exert its influence on identity preview benefit in the same processing level of semantics (i.e., N400, LPC).

## Materials and methods

### Participants

Native speakers of Chinese (*N* = 24; 8 males; ages 21–28) participated in the experiment. All participants were right-handed and reported normal or corrected-to-normal visual acuity. After data collection, one participant was removed because of excessive data loss (lack of items in one condition). This study was approved by the Psychology Research Ethics Committee of South China Normal University and participants were provided with written informed consent prior to the experiment.

### Materials

**Figure 1:**
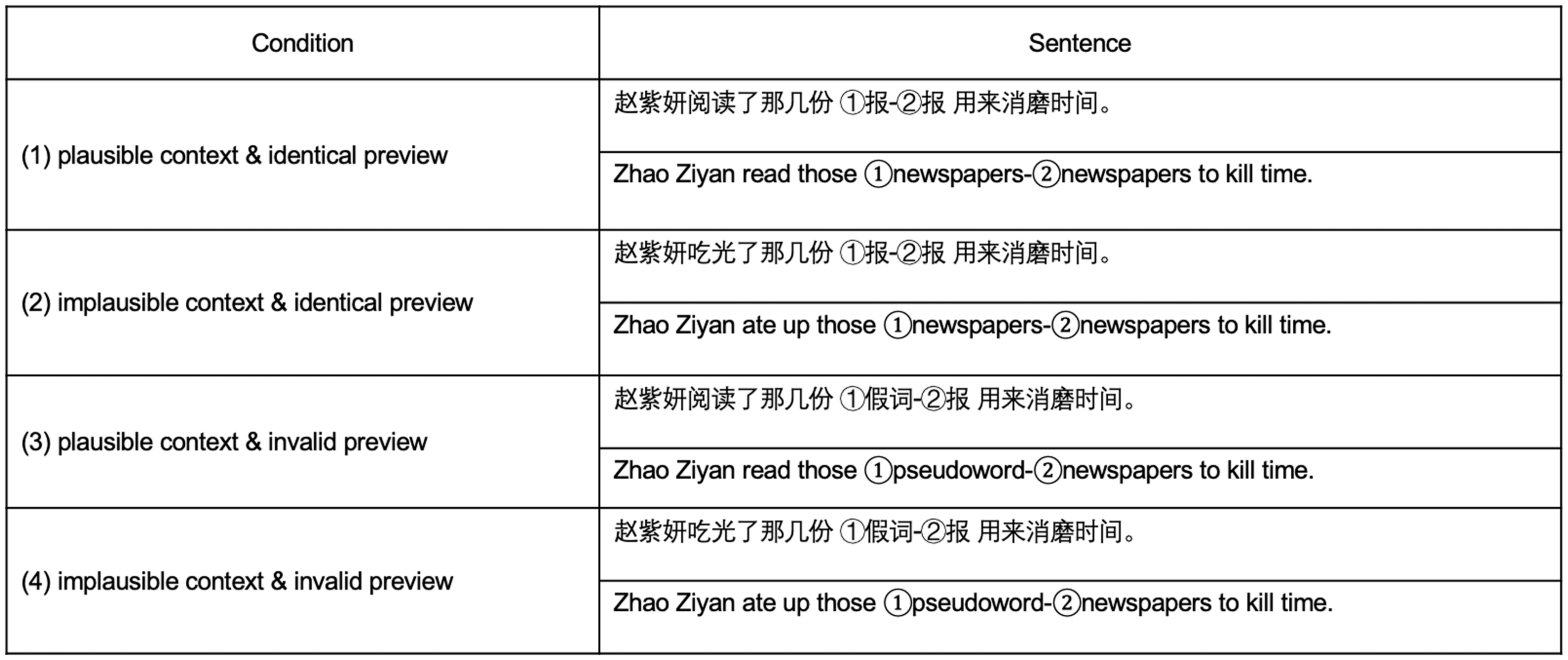
Example material and Condition. The target word was either plausible (read…newspapers) or implausible (ate up…newspapers) in the context of the sentence, and the target word either was presented in preview (newspapers-newspapers) or not present in preview (here: pseudoword-newspapers). These manipulations resulted in a 2 × 2 design (context plausibility × preview identity). The target word was always a single Chinese character. ① Target word in the preview position. ② Target word in the later foveal position.

We constructed 200 Chinese sentences with target words embedded near the middle position of the sentence. The sentences were between 13 and 20 characters in length (*M* = 16). In all sentences, the target word was a one-character word but the remaining words could be one, two, or three characters. The verb in each sentence was manipulated so that the target word was either plausible or implausible. For example, “newspaper” is plausible in the sentence “Zhao Ziyan *read* those newspapers to kill time,” and implausible in the sentence “Zhao Ziyan *ate up* those newspapers to kill time.” The strokes of the target word averaged 9.16 (SD = 3.39), and the frequency of the target word averaged 116.97 (SD = 478.71) per million (Cai and Brysbaert, 2010). The strokes of verbs averaged 17.54 (SD = 4.09) for the plausible condition and 17.76 (SD = 4.26) for the implausible condition. The frequency of verbs averaged 13.00 (SD = 26.25) per million for the plausible condition and 12.20 (SD = 27.95) per million for the implausible condition. The two types of verbs were matched in number of strokes, *t*(199) = 0.29*, p* = 0.769, and word frequency, *t*(199) = 0.53, *p* = 0.591. We also manipulated the preview of the target word so that it was either the same as the target word or a pseudoword. These manipulations resulted in four conditions: (1) plausible context & identical preview; (2) implausible context & identical preview; (3) plausible context & invalid preview; (4) implausible context & invalid preview (see Figure 1).

We asked 16 undergraduate students who did not participate in the main experiment to rate the plausibility of the sentence up to and including the target word. The students rated each sentence on a 5-point scale (1 = highly implausible; 5 = highly plausible). The mean rating for words in the plausible sentences was 4.56 (SD = 0.45), and the mean rating for words in the implausible sentences was 1.44 (*SD* = 0.47). These two values were significantly different from each other, *b* = 3.17, *SE* = 0.04, *t*(199) = 66.96, *p* < 0.001.

We asked 30 undergraduate students who did not participate in the main experiment and did not provide plausibility ratings to complete a sentence completion task (cloze procedure) to assess sentential constraint. Participants were presented with each sentence up to the target word (but not including it). They were then asked to complete the sentence with “the first word that comes to mind.” At the target word position, the contextual constraint averaged 0.41 (*SD* = 0.22) for sentences in which the target word was plausible, and it averaged 0.42 (*SD* = 0.23) for sentences in which the target word was implausible. These values were not significantly different from each other, *t*(199) = 0.40, *p = 0.682.*

### Procedure

Four sets of stimulus materials were created. Each set was made up of 200 sentences. The sentences were counterbalanced so that each condition of the same sentence frame appeared once across the four sets. After the study was described, the participants provided informed consent. By random assignment, each participant was assigned to one of four stimulus sets.

**Figure 2:**
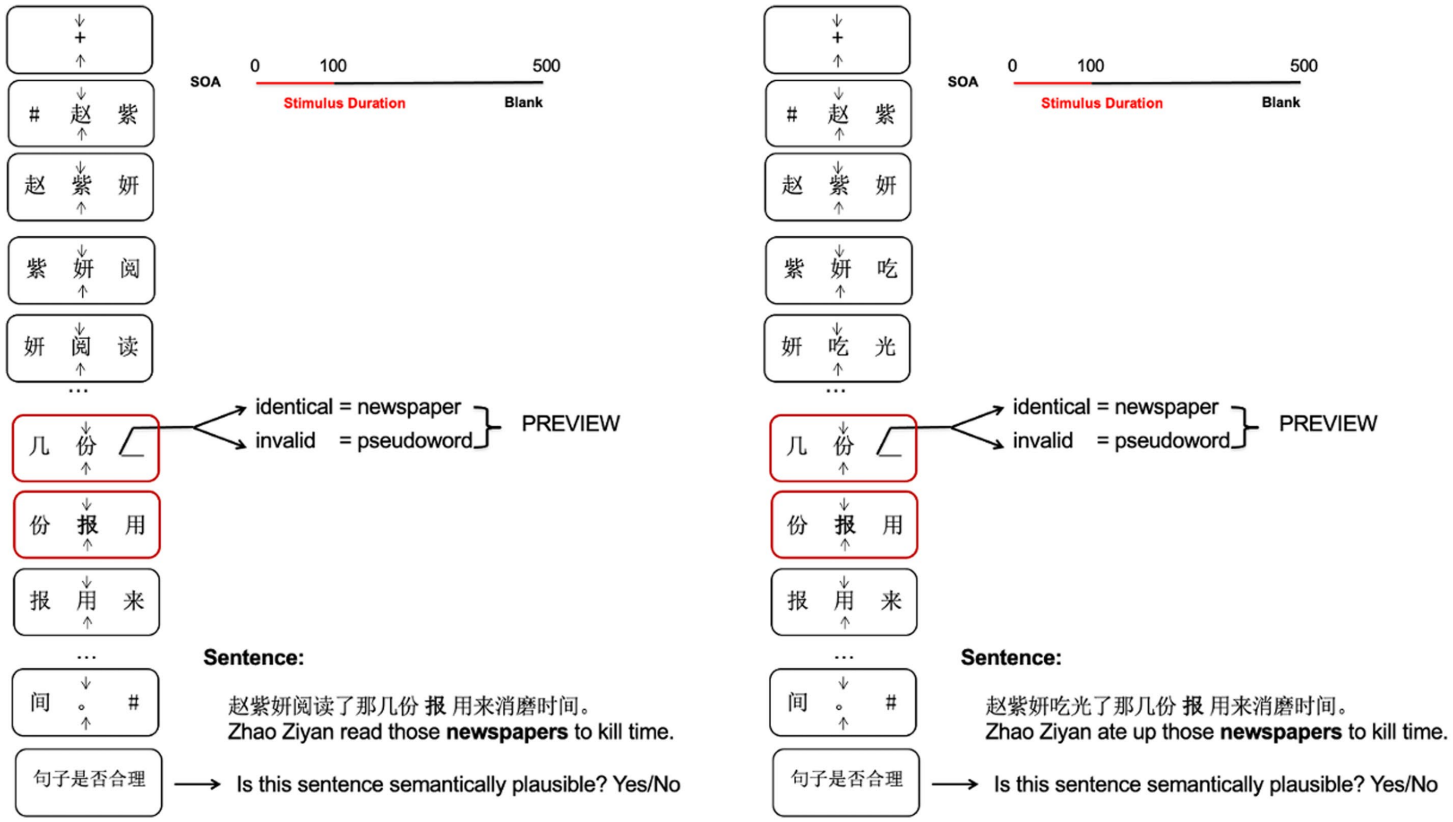
Trial scheme for the experiment. Sentences were presented character-by-character in the center of the screen, flanked by the preceding character to the left and the subsequent character to the right. Character triads were displayed for 100 ms each and separated by 400 ms blank intervals. The preview word position was presented either in its identity version (newspapers) or its invalid version (pseudoword) when the sentence was plausible (left side of the figure) and implausible (right side of the figure). Participants were asked to maintain their gaze on the central character (monitored with eye-tracking). They were also asked to judge the plausibility of the sentence with a button press at the end of the trial.

We adopted the RSVP-with-flankers paradigm to present the sentences. In this paradigm, to avoid eye movements, sentences were presented as triplets of characters during steady fixation. Each trial began with a fixation check. Then, sentences were presented character-by-character at the centre of the screen, flanked by the preceding character to the left and the subsequent character to the right. The triplets of characters were updated step-wise. Therefore, each character appeared in preview before it moved into foveal vision. The target preview position was presented either in its identity version (newspapers) or its invalid version (pseudoword). Triads of Chinese characters were displayed for 100 ms each and separated by 400 ms blank intervals (e.g., Barber et al., 2010). Participants were asked to maintain their gaze on the central character (monitored with eye-tracking). After each sentence, the participant was prompted to decide whether the sentence was semantically plausible and to indicate their decision by pressing either the left or right mouse button (see Figure 2).

Stimuli were displayed on a 17-inch CRT monitor (1,024 × 768 pixel resolution, vertical refresh 150 Hz). Each character subtended 1.62° × 1.62° degrees of visual angle. Characters were separated by an empty space the size of one character. Thus, the left edge of the right parafoveal flanker character was presented at an eccentricity of 2.43° (1.5 character spaces × 1.62°) from the screen center.

### Recordings and data analysis

Movements of the right eye were recorded with an SR Eyelink 1,000 eye-tracker (remote configuration) at 1000 Hz, with head position stabilized by a chin rest. Central fixation was verified gaze-contingently at the onset of each trial. The EEG was recorded from 42 Ag/AgCl electrodes mounted in a textile cap. The electrodes were placed at standard positions in the 10–10 electrode system. The electrooculogram was recorded from four electrodes positioned on the infraorbital ridge and outer canthus of each eye. The online reference was the left mastoid and FCz was used as the ground. Electrode impedances were kept below 5 kΩ. Signals were amplified using Brain Products at a time constant of 10s, and they were sampled at 500 Hz. Gaze and EEG were synchronized using the EYE-EEG extension (Dimigen et al., 2011) for EEGLAB (Delorme and Makeig, 2004). The EEG was high-pass filtered at 0.1 Hz (5th order Butterworth filter) and low-pass filtered at 45 Hz (using EEGLAB’s windowed sinc FIR filter with default settings). Finally, all channels were recalculated to average reference.

Overall, 30% of trials were excluded due to incorrect manual responses, EEG artifacts, or gaze samples outside the central fixation area while the target screen was presented. After the exclusion of all bad trials, a total of 3,182 trials remained (Cond1: 883, Cond2: 723, Cond3:840, Cond4:736). The EEG epochs of accepted trials were segmented around the onset of the parafoveal target word, from −200 to 1,500 ms, and baseline-corrected by subtracting a 100 ms pre-stimulus baseline. We defined an ROI of four occipitotemporal electrodes (PO9, PO7, PO8, PO10) to test the early preview positivity (e.g., Dimigen et al., 2012; Li et al., 2015, 2022; Antúnez et al., 2021), and an ROI of four centroparietal locations (Cz, Pz, CP1, CP2) to test the N400 and the LPC component (e.g., Dimigen et al., 2012; Li et al., 2015, 2022; Antúnez et al., 2021). The time windows of 150–250 ms, 250–450 ms and 500–800 ms after the onset of the targets in the foveal position were defined to test the preview positivity, N400, and LPC components, respectively (e.g., Grainger et al., 2006; Dimigen et al., 2012; Rommers and Federmeier, 2018; Antúnez et al., 2021).

Single-trial ERP amplitudes were calculated as the average amplitude in the ROI across the two specified time windows. Statistical analyses were performed using linear mixed effect models (LMM). LMM offer the option to specify participants and items as crossed random factors, and can handle imbalanced datasets due to missing data without losing statistical power (Pinheiro and Bates, 2000). We defined two fixed factors: (1) *contextual plausibility* (plausible, implausible) and (2) *preview word identity* (identical with the target word, pseudoword). Participants and items were included as crossed random factors. The maximum random effects structure that included random intercepts and slopes of fixed factors (contextual plausibility and preview identity) did not converge, so a simple random effects structure with random slopes removed was used to obtain convergence.

### Results

On average, participants correctly answered the question after 86% of the trials, indicating that the participants correctly understood most sentences. Response accuracy was significantly lower in the implausible conditions (80%) than in the plausible conditions (93%), *b* = 1.34, *SE* = 0.10, *t* = 13.36, *p* < 0.001.

### Identity preview effect

**Figure 3:**
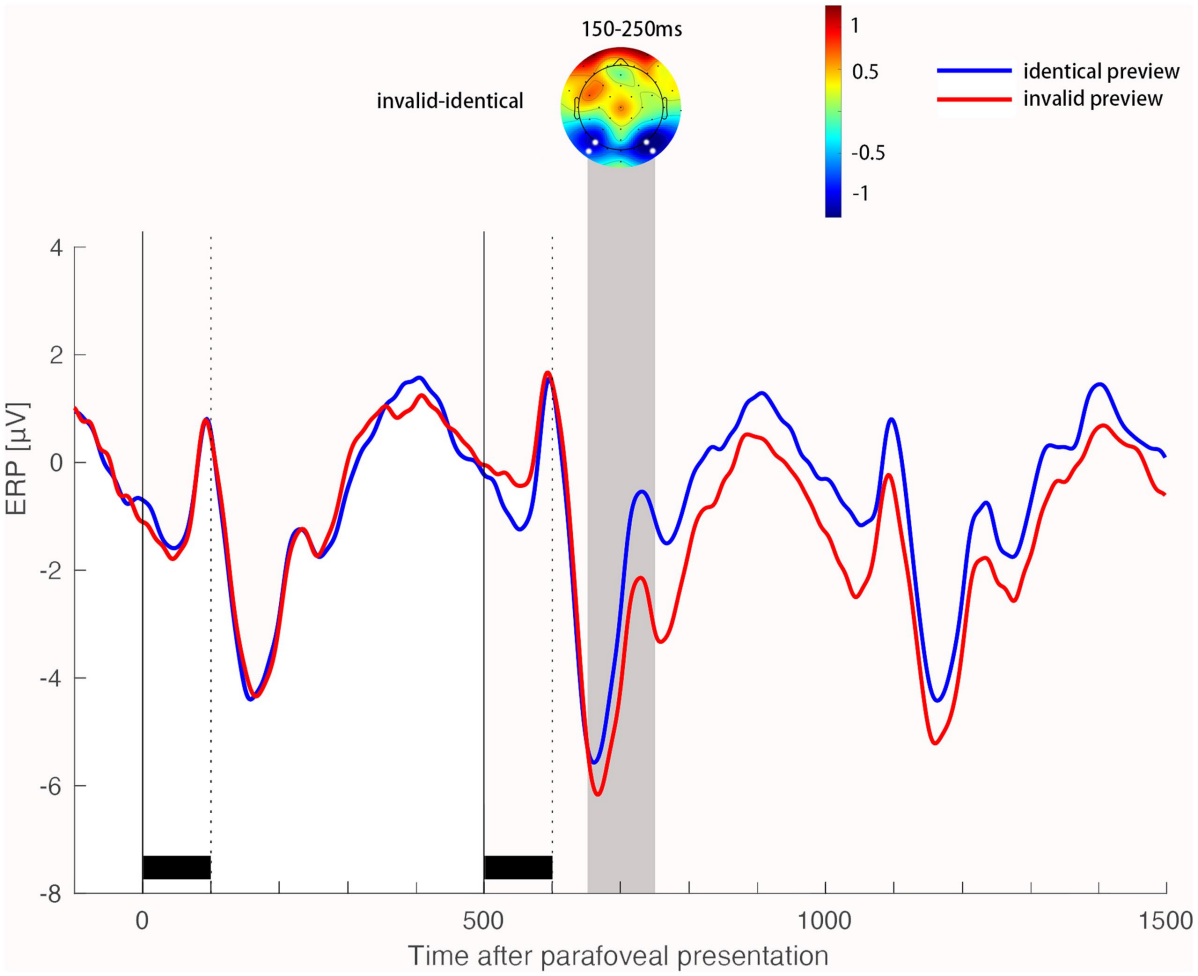
Main effect of preview identity on the early preview positivity at occipitotemporal electrodes. Time 0 on the time axis marks the onset of the target word in the right parafoveal position. Black bars above the time axis mark the stimulus durations of the character triads. The two vertical lines mark the onset of the triad containing the target word in the parafovea and fovea, respectively. ERP waveforms show the average activity in the ROI of four electrodes (highlighted in white in the topographic map) for the identical preview condition and invalid preview condition. Scalp topographic map shows the difference in ERP activity (invalid preview conditions minus identical preview conditions) in the time window of 150–250 ms after the onset of the word in the foveal position.

The identity preview effect was tested on the preview positivity, N400 and LPC components locked to the onset of the target word in fovea. As shown in Figure 3, we found the identity preview effect influence the preview positivity. An identical parafoveal preview elicited a greater positivity than an invalid parafoveal preview in the occipitotemporal region 150-250 ms after the onset of the target word in fovea, *b* = 1.24, *SE* = 0.22, *t* = 5.62, *p* < 0.001. This early identity preview effect did not interact with the contextual plausibility, *b* = 0.15, *SE* = 0.44, *t* = 0.35, *p* = 0.726.

In the N400 time window, we found the identity preview effect interacted with the contextual plausibility in the centroparietal region 250-450 ms after the onset of the target word in fovea, *b* = 0.91, *SE* = 0.45, *t* = 2.01, *p* = 0.044. Specifically, an invalid parafoveal preview elicited a more negative N400 component than an identical parafoveal preview in the plausible sentence (*b* = 0.96, *SE* = 0.30, *t* = 3.16, *p* = 0.002), but not in the implausible sentence (*b* = 0.05, *SE* = 0.33, *t* = 0.18, *p* = 0.861).

**Figure 4:**
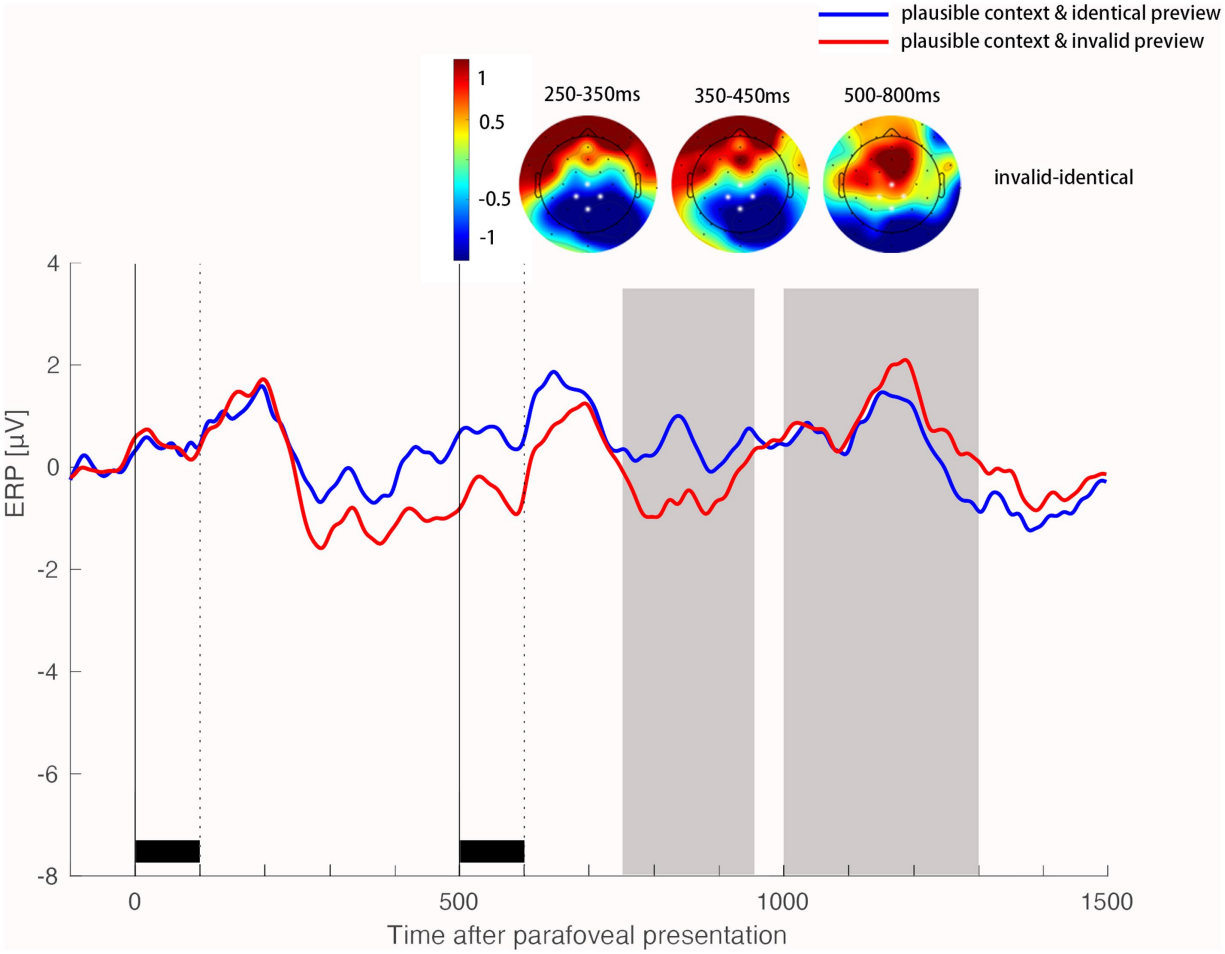
Identity preview benefit on the N400 and LPC components when the target word was plausible in the sentence. Time 0 on the time axis marks the onset of the target word in the right parafoveal position. ERP waveforms show the average activity in the ROI of four electrodes (highlighted in white in the topographic map) for the identical preview condition and invalid preview condition. Scalp topographic maps show the difference in ERP activity (invalid preview conditions minus identical preview conditions) in the time window of 250–350 ms, 350–450 ms and 500–800 ms after the onset of the target word in the foveal position, respectively.

**Figure 5:**
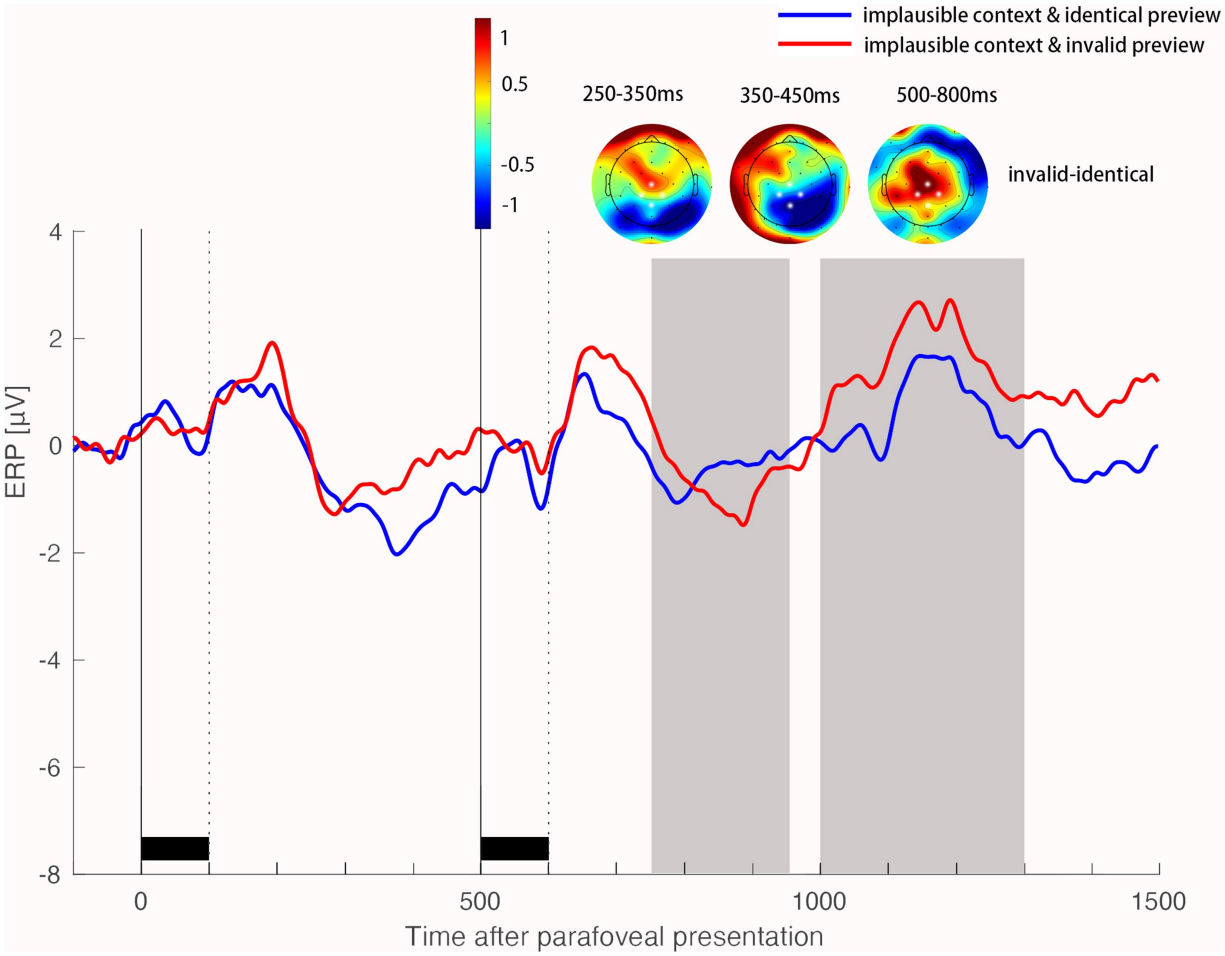
Identity preview benefit on the N400 and LPC components when the target word was implausible in the sentence. Time 0 on the time axis marks the onset of the target word in the right parafoveal position. ERP waveforms show the average activity in the ROI of four electrodes (highlighted in white in the topographic map) for the identical preview condition and invalid preview condition. Scalp topographic maps show the difference in ERP activity (invalid preview conditions minus identical preview conditions) in the time window of 250–350 ms, 350–450 ms and 500–800 ms after the onset of the target word in the foveal position, respectively.

In addition, we found that this interaction appeared in the early N400 window (250-350 ms), but not in the late N400 window (350–450 ms; Figures 4, 5). Specifically, in the early N400 window (250-350 ms), the identity preview effect interacted with the contextual plausibility (*b* = 1.42, *SE* = 0.46, *t* = 3.07, *p* = 0.002). An invalid parafoveal preview elicited a more negative N400 component than identical parafoveal preview in the plausible sentence (*b* = 1.06, *SE* = 0.31, *t* = 3.39, *p* < 0.001) but not in the implausible sentence (*b* = 0.35, *SE* = 0.34, *t* = 1.04, *p* = 0.294). In the late N400 window (350-450 ms), we found a main effect of preview identity, *b* = 0.68, *SE* = 0.23, *t* = 2.83, *p* = 0.004. This late identity preview effect did not significantly interact with the contextual plausibility, *b* = 0.38, *SE* = 0.47, *t* = 0.81, *p* = 0.417.

**Figure 6:**
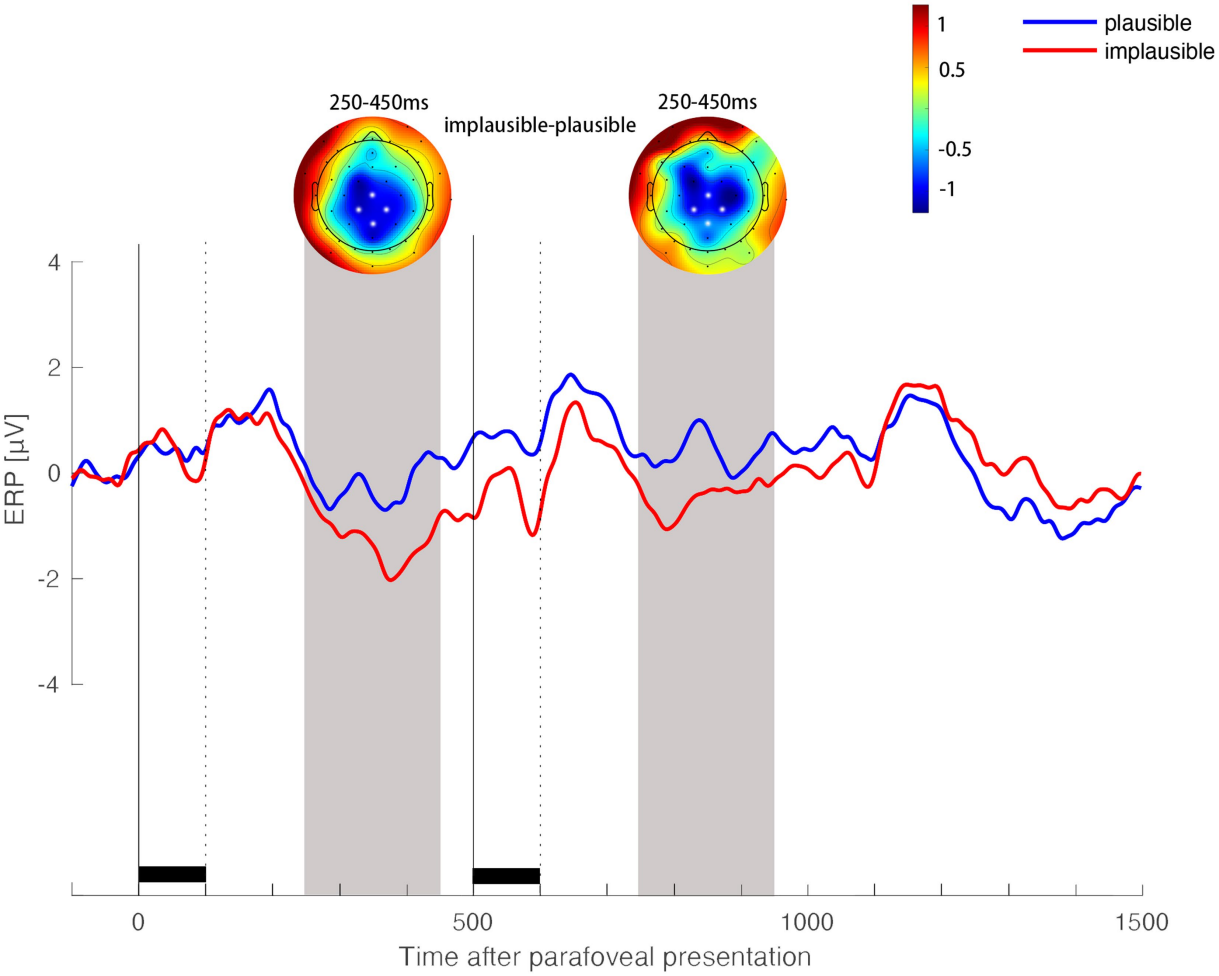
ERP plausibility effects when the target word appeared in the parafovea and subsequently again in the fovea. ERP waveforms show the average activity in the ROI of four centroparietal electrodes (highlighted in white in the topographic map) for the plausible context & identical preview condition and implausible context & identical preview condition. Scalp topographic maps show the difference in ERP activity (plausible context & identical preview minus implausible context & identical preview) in the time window of 250–450 ms after the onset of the word in the parafoveal and in the foveal position, respectively.

In the late LPC time window, we found a main identity preview effect. An identical parafovea preview elicited a more positive LPC component than an invalid parafovea preview in the centroparietal region 500–800 ms after the onset of the target word in fovea, *b* = 0.81, *SE* = 0.24, *t* = 3.31, *p* < 0.001. This late identity preview effect did not interact with the contextual plausibility, *b* = 0.85, *SE* = 0.48, *t* = 1.75, *p* = 0.080.

### Contextual plausibility effect

It is valuable to ensure that the contextual conflict indeed exists in readers’ on-line sentence processing. It is the premise for further investigation of the impact of contextual conflict on the identity preview priming process. Therefore, we tested the contextual plausibility effect by comparing the processing of target word in the plausible context condition (Condition 1 plausible context & identical preview) with the processing of target word in the implausible context condition (Condition 2 implausible context & identical preview). The N400 effect of the target word’s plausibility appeared in the centroparietal region 250-450 ms after the onset of the target word in parafovea, *b* = 1.22, *SE* = 0.26, *t* = 4.71, *p* < 0.001. Implausible parafoveal words elicited a more negative waveform than plausible parafoveal words. This plausibility effect extended to the foveal N400 time window in the centroparietal region 250-450 ms after the onset of the target word in fovea, *b* = 1.03, *SE* = 0.31, *t* = 3.26, *p* = 0.001 (see Figure 6).

## Discussion

In the present study, we aim to investigate the repetition effect due to a preview word in a consistent and in a conflicting sentential context. Although the identity preview benefit has been confirmed by a large number of studies (Rayner, 1998, 2009), the mechanism of the identity preview benefit, the level of lexical processing it occurs, and its relationship to the sentential context remain unclear. We conducted an ERP experiment with Chinese readers using the RSVP-with-flankers paradigm and rigorous fixation control *via* eye tracking. We manipulated the sentential context to make the target word plausible or not plausible with the sentence and manipulated the target word present or not present in preview. We found identity preview repetition can trigger a cascade of processes involving the word in fovea, including the early preview positivity, N400 and LPC. Contextual conflicts can interfere with this repetition process on the N400 components. In the following, these results are discussed in turn.

First, the repetition of a preview word leads to a greater preview positivity, and a reduction of the N400, and an amplification of the LPC. The results suggest that the repetition priming effect of preview can affect the multiple processes in fovea. The early preview positivity may reflect orthographic processing of the word (e.g., Dimigen et al., 2012). The late effects of preview validity on the N400 may reflect a form of repetition priming at the level of semantics (e.g., Kutas and Federmeier, 2011), and the LPC may be linked to explicit recall of the prior presentation and context updating (e.g., Paller et al., 1995). This suggests that the word in preview can fully activate/prime the foveal word at multiple levels. This repetition priming process from preview is very similar to the effect of repetition from a previously fixated word (e.g., Rugg, 1985; Holcomb and Grainger, 2007). Thus, immediate and in-depth activation/priming does not necessarily need to be processed consciously, nor will it be eliminated due to the limitation of visual acuity. This suggests that the reader is actively using the information available to complete the language process efficiently.

Second, we found that the repetition effect from a preview word can be disrupted by contextual conflict, evidenced by the early N400 components. When the contextual conflict occurred, the identity preview effect was manifested only in the late semantic time window (350–450 ms), but not in the early semantic time window (250–350 ms). Now we get a view of the process by which contextual conflict interferes with the repetition effect. The preview effect on the preview positivity may reflect processing of the word at the primary level of orthography (Dimigen et al., 2012). Our results showed that this low-level lexical process was not easily affected by the higher level of contextual information. In the initial stage of semantic processing of target words, however, the preview priming was disturbed by the contextual conflict. This suggests that word processing at the semantic level is jointly influenced by semantic information from both preview priming words and sentence context. However, the contextual interruption on the priming effect was short-lived and did not persist to the late semantic processing of the foveal word.

Why do contextual conflicts interfere with the repetition priming from a preview word? There are several possible reasons. One possibility is that the meaning conflict of the context delays the repetition priming from a preview word. This delay may be related to a resource limitation of the cognitive process. The semantic integration of a word into an implausible context is an effortful, resource-limited process. Hence, it may be difficult to start the semantic priming within a short interval because the semantic system may be refractory after the processing of the implausible word. Such resource limitations for semantic processes have been reported for the N400 elicited by word pairs (Hohlfeld et al., 2004). Another possibility is that the context conflict inhibits the preview repetition priming at a certain level of processing. Some previous studies (e.g., Barber et al., 2002; Domínguez et al., 2004) suggested that N400 modulation could reflect two subcomponents: an early component (250–350 msec) related to morpho-decomposition at the lexeme level and a later component (after 350 msec) related to lexical and meaning selection at the lemma level. Contextual conflict may inhibit preview priming in early morpho-orthographic stages, but not in late morpho-semantic stages. The present results indicate that the contextual conflict can change the repetition process at the semantic level.

Contextual conflict can affect the repetition effect from the word in preview, consistent with the episodic accounts and the discourse model of word processing. Thus, it is important to consider the global context in the repetition process during sentence reading. Our results also showed that the effect of contextual conflict on repetition process was very rapid and short-lived, and only appeared in the early semantic processing window when the repetition occurred rapidly. This partly explains why the interruption of contextual conflict has not been observed in previous studies of priming from the fixated word. Priming from a preview word happens more quickly, while priming from a previously fixated word happens more slowly. Future research may need to consider the priming occurring at which speed is most sensitive to changes in contextual information.

## Conclusion

Our study found that repetition of a preview word can trigger a cascade of processes involving the word in fovea. These processes involve the early preview positivity reflecting the early orthographic processing of words, and the N400 and LPC components, reflecting late semantic processing of words. Contextual conflicts can temporarily interfere with the identity preview effect at the semantic level, indicating that the processing of a word is affected by the in-depth and immediate impact of contextual information.

## Data availability statement

The original contributions presented in the study are included in the article/supplementary material, further inquiries can be directed to the corresponding author.

## Ethics statement

The studies involving human participants were reviewed and approved by the Psychology Research Ethics Committee of South China Normal University. The patients/participants provided their written informed consent to participate in this study.

## Author contributions

NL and SW: conceptualization and writing—review and editing. GL and NL: investigation, methodology, and writing—original draft. NL: funding acquisition. All authors contributed to the article and approved the submitted version.

## Funding

This work was supported by the National Natural Science Foundation of China (grant: 31700992) and the National Social Science Fund of China (grant: 21BYY099). The funders had no role in study design, data collection and analysis, decision to publish, or preparation of the manuscript.

## Conflict of interest

The authors declare that the research was conducted in the absence of any commercial or financial relationships that could be construed as a potential conflict of interest.

## Publisher’s note

All claims expressed in this article are solely those of the authors and do not necessarily represent those of their affiliated organizations, or those of the publisher, the editors and the reviewers. Any product that may be evaluated in this article, or claim that may be made by its manufacturer, is not guaranteed or endorsed by the publisher.
